# Multifractal Analysis of Ion-Channel Currents: A Comparative Study of the WTMM and MFDFA Approaches

**DOI:** 10.3390/membranes16080261

**Published:** 2026-08-04

**Authors:** Janusz Miśkiewicz, Zbigniew Burdach, Agnieszka Siemieniuk, Waldemar Karcz

**Affiliations:** 1 Institute of Theoretical Physics, University of Wrocław, 50-204 Wrocław, Poland; 2 Physics and Biophysics Department, Wrocław University of Environmental and Life Sciences, 50-375 Wrocław, Poland; 3 Faculty of Natural Sciences, Institute of Biology, Biotechnology and Environmental Protection, University of Silesia in Katowice, 40-032 Katowice, Poland; zbigniew.burdach@us.edu.pl (Z.B.); agnieszka.siemieniuk@us.edu.pl (A.S.); waldemar.karcz@us.edu.pl (W.K.)

**Keywords:** SV channel, patch clamp, multifractal analysis, WTMM, time series analysis, long memory effect

## Abstract

The multifractal properties of ion-channel currents recorded from *Beta vulgaris* L. vacuoles were analyzed using multifractal detrended fluctuation analysis (MFDFA) and the Wavelet Transform Modulus Maxima (WTMM) method. The study covered a range of membrane potentials, different incubation times with indole-3-acetic acid (IAA), and regimes with single and multiple active channels. Clear multifractal behavior was observed, with the spectrum width, Δα, varying with voltage, biochemical modulation, and channel occupancy. A comparative analysis shows that WTMM provides a more reliable and detailed characterization of the scaling properties. In particular, WTMM yields stronger nonlinearity in the scaling exponents τ(q), clearer scaling regimes, and higher sensitivity to variations in Δα, whereas MFDFA results are affected by deviations from ideal scaling. The presence of multiple channels introduces a structured, non-monotonic dependence of Δα on voltage, indicating collective dynamics. These results demonstrate that WTMM is better suited for analyzing nonstationary, dichotomous ion-channel signals.

## 1. Introduction

A broad range of complex systems presents sophisticated multifractal properties. Multifractal properties are observed in physical systems such as turbulent flow, flow through porous media, fracture surfaces of metals or diffusion-limited aggregation (DLA) growth [1,2]; climate variability–temperature fluctuations, drought dynamics, precipitation records and others; and in seismology and geophysical analysis, e.g., geomagnetic fields and seismically active regions [3,4]. Multifractal features have also been observed in financial time series analysis [5,6]. Multifractal analysis was even successfully applied in text analysis [7]. Another area of multifractal analysis pertains to biology and physiological signals. Network physiology and multifractal fluctuation analysis jointly provide new biomarkers for disease diagnosis and homeostatic dysregulation [8]. This study focuses on the properties of ion-channel currents. Recent work demonstrates that ion currents in various cell types exhibit multifractal features [9,10,11,12]. It should be noted that the authors use multifractal detrended fluctuation analysis (MFDFA) in their study, which is considered a standard in those analyses. The method is described in Section 3.1. The alternative method of multifractal analysis is the wavelet-based method, i.e., wavelet modulus maxima (WTMM) [13,14,15] and wavelet leaders. In this paper, the WTMM methods will be used. The key elements of WTMM methods are shortly presented in Section 3.2. It relies on the wavelet transform to decompose a signal into localized components at multiple scales, allowing one to probe singularities (local regularity) in a robust, noise-resistant way. By studying the scaling behavior of wavelet coefficients—or wavelet modulus maxima—one can extract scaling exponents and construct the multifractal spectrum, which quantifies the distribution of Hölder exponents in the signal. The fundamental difference between the MFDFA and WTMM methods is that the former assumes a linear (or, more generally, polynomial) evolution of the signal, so the fluctuation in the quality of fit is analyzed across different scales, whereas the latter is based on wavelet analysis. In many applications, both methods are considered equally reliable [13,16,17,18]. However, these methods are not equivalent. Comparative studies have shown that, particularly for finite and noisy datasets, the estimated multifractal spectrum may depend on the chosen formalism, scaling range, and estimator, leading to noticeable method-dependent differences [16,17,19,20]. In the present study, we therefore compare the results from the MFDFA method with those obtained from the WTMM formalism using the Haar wavelet. This wavelet was chosen because of the fundamental structure of the ion-channel signal feature: a dichotomous signal that switches the current magnitude between *closed* and *open* states.

Within this study, the *Beta vulgaris* L. taproot vacuoles, the slowly activating vacuolar (SV) channel ion current multifractal features, and the influence of IAA (indole-3-acetic acid) are investigated. The slowly activating vacuolar (SV) channels correspond to the tonoplast-localized TPC1 (Two-Pore Channel 1) and represent the dominant voltage-dependent cation conductance of plant vacuoles. These channels are non-selective for cations (e.g., K^+^, Na^+^, Ca^2+^) and are activated by membrane depolarization and cytosolic Ca^2+^, while being inhibited by luminal Ca^2+^ and modulated by pH, redox state, and polyamines. SV channels are encoded by *TPC1* and are widely recognized as key components of vacuolar excitability and Ca^2+^-dependent signaling networks in plant cells [21].

IAA (indole-3-acetic acid), the principal auxin in plants, is a central regulator of plant growth and development, controlling cell expansion, division, and differentiation through transcriptional (TIR1/AFB-mediated) and rapid non-transcriptional signaling pathways [22].

Importantly, SV channel activity is modulated by IAA. Experimental evidence in plant vacuoles shows that physiologically relevant concentrations of IAA can alter the amplitude and kinetics of SV current, indicating that auxin signaling affects tonoplast ion transport. In *Beta vulgaris* L. taproot vacuoles, IAA has been reported to modulate SV channel activity, suggesting involvement of downstream signaling cascades that link hormonal perception to vacuolar electrical properties [23]. More recent syntheses emphasize that TPC1-mediated SV currents participate in cellular stress signaling by coupling K^+^ and Ca^2+^ fluxes with environmental and metabolic cues [24,25].

Overall, SV/TPC1 channels act as integrative hubs combining voltage-, Ca^2+^-, and auxin-dependent regulation, thereby linking vacuolar ion homeostasis with hormonal control of plant growth and adaptive responses.

The paper is organized as follows: in Section 2, the experimental details are described; in Section 3, the necessary background on multifractal methods is presented. The results of the multifractal analysis are presented in Section 4.

## 2. Materials and Methods

Electrophysiological measurements were performed using the patch-clamp technique in an excised cytosolic side-out configuration. Currents were recorded with an EPC-7 Plus amplifier (List-Medical-Electronic, Darmstadt, Germany), as previously described [23]. Signals were filtered using a five-pole Bessel filter and sampled at frequencies between 1 and 100 kHz.

Patch pipettes were fabricated from borosilicate glass capillaries (Kimax-51, Kimble Products, Toledo, OH, USA) according to standard procedures. Access to the vacuolar interior was achieved by applying voltage pulses in the range of 500–900 mV combined with gentle suction. Current and voltage conventions followed those described by Bertl et al. [26].

Vacuoles from taproots of Beta vulgaris were isolated using a previously described mechanical method [23]. The standard bath solution contained 100 mM K^+^ (100 mM KCl), 2 mM MgCl_2_, 0.1 mM CaCl_2_, 2 mM DTT, 5 mM MES, 5 mM Tris, and 400 mM sorbitol, adjusted to pH 7.5 and an osmolality of 650 mOsm. Pipettes were filled with a solution containing 100 mM KCl, 2 mM MgCl_2_, 5 mM MES, and 5 mM Tris (pH 5.5), with osmolality adjusted to 580 mOsm using sorbitol.

The concentration of IAA (1 μM) was selected as the most effective for SV channel activity among those tested (0.1, 1, and 10 μM). For treatment, the control bath solution was replaced with an identical solution supplemented with 1 μM IAA. Solution exchange was achieved by continuous perfusion of the recording chamber using an SP200 infusion pump (World Precision Instruments, Sarasota, FL, USA). All experiments were conducted at room temperature (22 ± 1 °C).

Data were acquired using PatchMaster (Version 1.7.0) and FitMaster (Version 2x92) software (HEKA Electronic, Lambrecht, Germany).

## 3. Mathematical Background

### 3.1. Multifractal Detrended Fluctuation Analysis (MFDFA)

Multifractal detrended fluctuation analysis is the generalization of the detrended fluctuation analysis (DFA) [20,27]. Its brief description is presented here for the reader’s convenience.

Let us denote the analyzed signal as x(i), i=1,…,N. The integrated profile is defined as

(1) $$Y\left(j\right)=\sum_{i=1}^{j}\left[x(i)-\langle x\rangle \right],\;j=1,\ldots ,N,$$

where 〈x〉 is the mean of the series. The profile is divided into $N_{d}=\left\lfloor N/d\right\rfloor$ non-overlapping segments of length *d*, and the procedure is repeated from the opposite end, yielding $2N_{d}$ segments. In each segment ν, a polynomial trend $y_{\nu }\left(j\right)$ of order *m* (usually linear) is fitted and removed, and the detrended variance is computed as

(2) $$F^{2}\left(\nu ,d\right)=\frac{1}{d}\sum_{j=1}^{d}{\left[Y\;\left((\nu -1)d+j\right)-y_{\nu }\left(j\right)\right]}^{2}.$$

The *q*-order fluctuation function is then defined by

(3) $$F_{q}\left(d\right)={\left\{\frac{1}{2N_{d}}\sum_{\nu =1}^{2N_{d}}{\left[F^{2}\left(\nu ,d\right)\right]}^{q/2}\right\}}^{1/q},\;q\neq 0,$$

while for q=0, logarithmic averaging is used. If scale invariance holds, the fluctuation functions obey a power-law relation

(4) $$F_{q}\left(d\right)∼d^{h(q)},$$

where h(q) is the generalized Hurst exponent. A nonlinear dependence of h(q) on *q* indicates multifractality. The multifractal mass exponent is given by

(5) τ(q)=qh(q)−1,

and the singularity spectrum f(α) is obtained via a Legendre transform:

(6) $$\alpha =\frac{d\tau (q)}{dq},\;f\left(\alpha \right)=q\;\alpha -\tau \left(q\right).$$

Here, α denotes the Hölder (singularity) exponent. The width and shape of f(α) provide a quantitative measure of the degree of multifractality.

### 3.2. Wavelet Transform Modulus Maxima Method

The Wavelet Transform Modulus Maxima (WTMM) method is a multifractal analysis technique based on the continuous wavelet transform (CWT), designed to characterize scaling properties and local singularities in nonstationary signals [28,29]. Similar to MFDFA, the method relies on the scaling behavior of fluctuation measures with respect to the observation scale *s* but uses wavelet coefficients to probe local regularity.

For a signal x(t), the continuous wavelet transform is defined as

(7) $$W_{x}\left(s,t\right)=\frac{1}{s}\int x\left(u\right)\;\psi \;\left(\frac{u-t}{s}\right)\;du,$$

where *s* denotes the scale, *t* the position, and ψ is an analyzing wavelet with a sufficient number of vanishing moments, typically chosen as a derivative of the Gaussian. The vanishing moments suppress polynomial trends, allowing the accurate detection of singular structures.

Local singularities are identified by the maxima of the wavelet modulus $|W_{x}\left(s,t\right)|$ along *t*. The connected set of these maxima across scales forms maxima lines, whose scaling behavior reflects the local Hölder exponent α of the signal [30].

The multifractal formalism is constructed from the partition function

(8) $$Z\left(q,s\right)=\sum_{\ell \in L(s)}{\left|W_{x}\left(s,t_{\ell }\right)\right|}^{q}∼s^{\tau (q)},$$

where the sum extends over all wavelet modulus maxima lines L(s) at scale *s*. The scaling exponents τ(q) are estimated from the power-law dependence of Z(q,s) on *s*. For one-dimensional signals, the exponents τ(q) are directly comparable to those obtained in MFDFA, where τ(q)=qh(q)−1.

The singularity spectrum f(α) is obtained via the Legendre transform

(9) $$\alpha =\frac{d\tau (q)}{dq},\;f\left(\alpha \right)=q\alpha -\tau \left(q\right).$$

**Figure 1:**
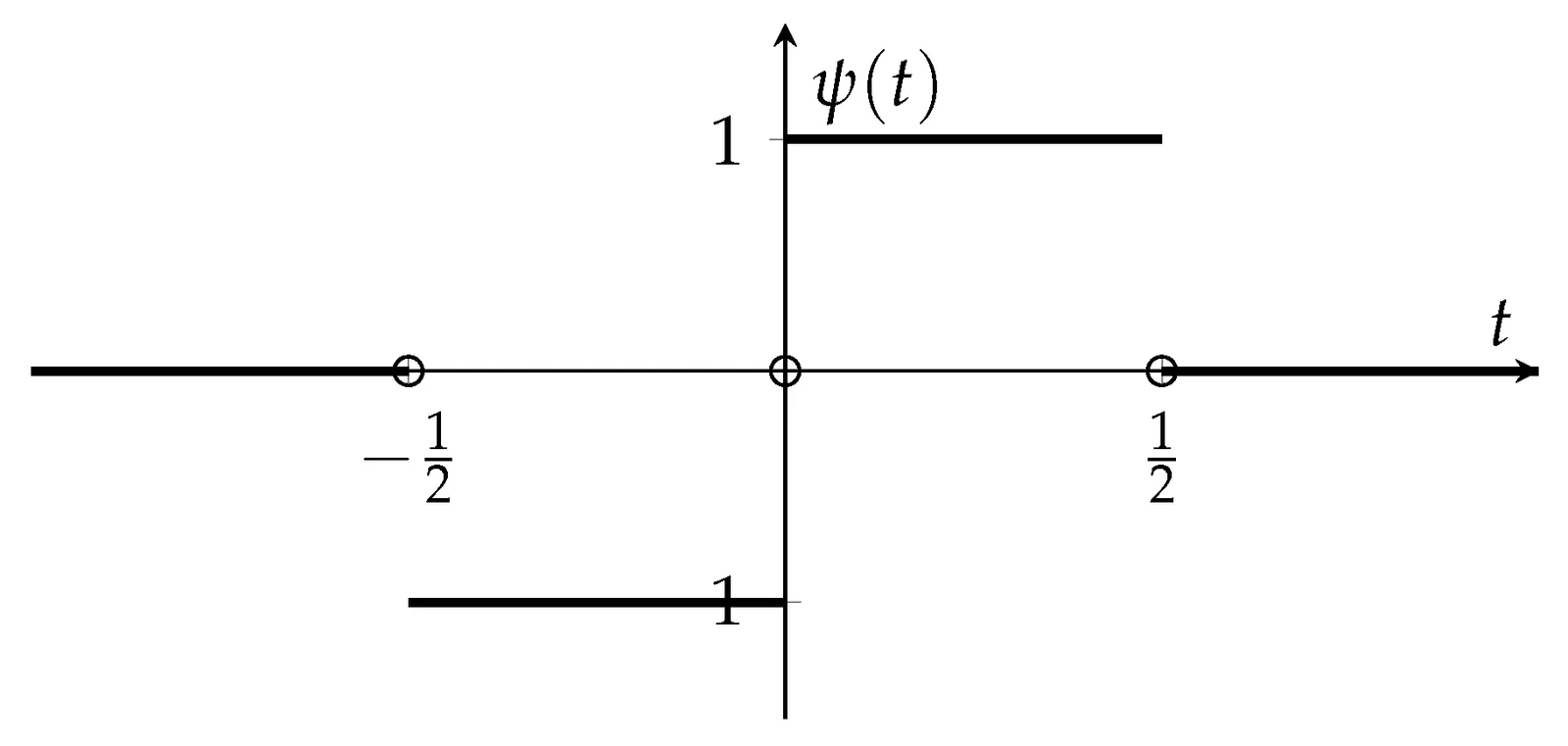
Haar wavelet ψ(t).

The WTMM method provides direct access to local singularities and is particularly effective for highly nonstationary signals encountered in turbulence, biology, and finance [13,31]. Its main drawbacks are higher computational cost and sensitivity to the choice of wavelet and maxima tracking procedure. In the WTMM framework, the analyzing wavelets are typically chosen as derivatives of the Gaussian function, due to their well-defined number of vanishing moments, which guarantees effective suppression of polynomial trends and high sensitivity to local singularities [28,29,30]. However, in the present study, the Haar wavelet (Figure 1) is used (Equation (10)) due to its perfect correspondence to the shape of the ion-channel dichotomous signal.

(10) $$\psi \left(t\right)=\left\{\begin{array}{cc} 1, & 0\leq t<\frac{1}{2},\\ -1, & -\frac{1}{2}\leq t<0,\\ 0, & \mathrm{otherwise}. \end{array}\right.$$

## 4. Results

### 4.1. Data

**Figure 2:**
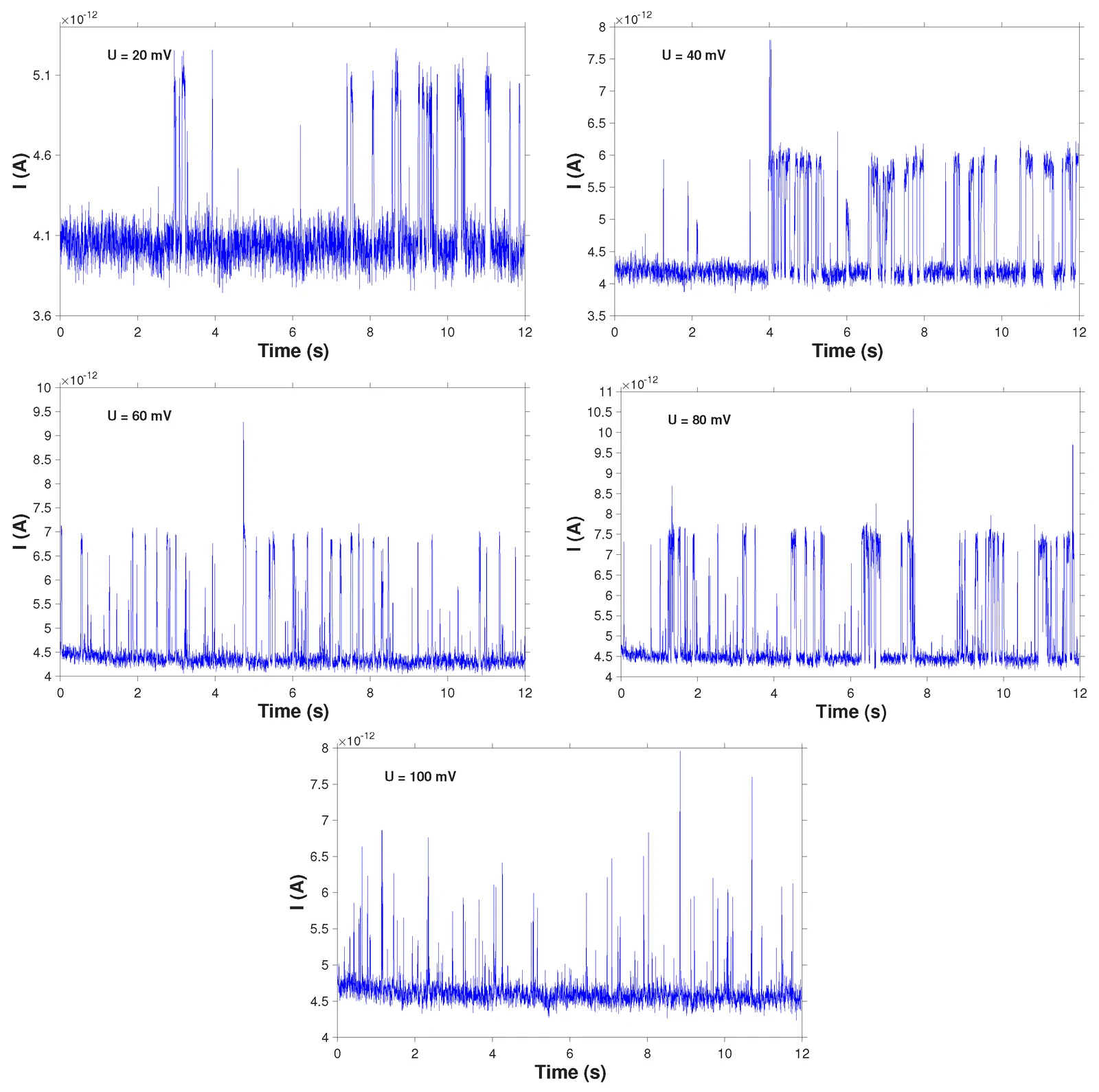
Examples of the registered ion currents at different membrane potentials. Control environment.

In this study, data were analyzed from patch-clamp recordings obtained at membrane potentials ranging from 10 mV to 100 mV in 20 mV steps. The signals were recorded with a sampling interval of 0.05 ms. Each time series consisted of 240,000 data points, corresponding to a recording duration of 12 s. Experiments were performed for the control condition and after incubation with IAA for 1, 5, and 15 min. Measurements were repeated, yielding 9–16 independent recordings for each condition (membrane potential and incubation time). Representative examples of the recorded current traces are shown in the figures below (Figure 2). The key feature to note is the characteristic shape of the ion current signal, which corresponds to the channel’s open and closed states.

### 4.2. Qualitative Comparison of MFDFA vs. WTMM

As discussed in the introduction, the main goal of the paper is to verify whether the WTMM method provides more reliable results. Therefore, all the data were analyzed by both methods. Let us focus on the chosen examples of outcome to discuss the main features: the control environment and 5 min incubation in IAA at 20 mV, 60 mV, and 100 mV. All the results are available on request from authors.

The numerical analyses were performed using MATLAB R2026a. For MFDFA, fluctuation functions $F_{q}\left(s\right)$ were calculated using 30 logarithmically spaced scales ranging from $s_{\min}=16$ to $s_{\max}=\left\lfloor N/4\right\rfloor$, where N denotes the signal length. Generalized Hurst exponents h(q) were estimated from linear regressions of $\log_{2}F_{q}\left(s\right)$ versus $\log_{2}s$ over the entire predefined scale interval.

For WTMM, a wavelet-leader approach based on the Haar wavelet was employed. The maximum dyadic scale was defined as $J=\lfloor \log_{2}N\rfloor$. Scaling exponents τ(q) were obtained from least-squares fits of $\log_{2}S\left(q,j\right)$ versus the dyadic scale index *j* using all available scales between j=1 and j=J.

Identical procedures were applied to all recordings, membrane voltages, incubation times, and surrogate datasets.

**Figure 3:**
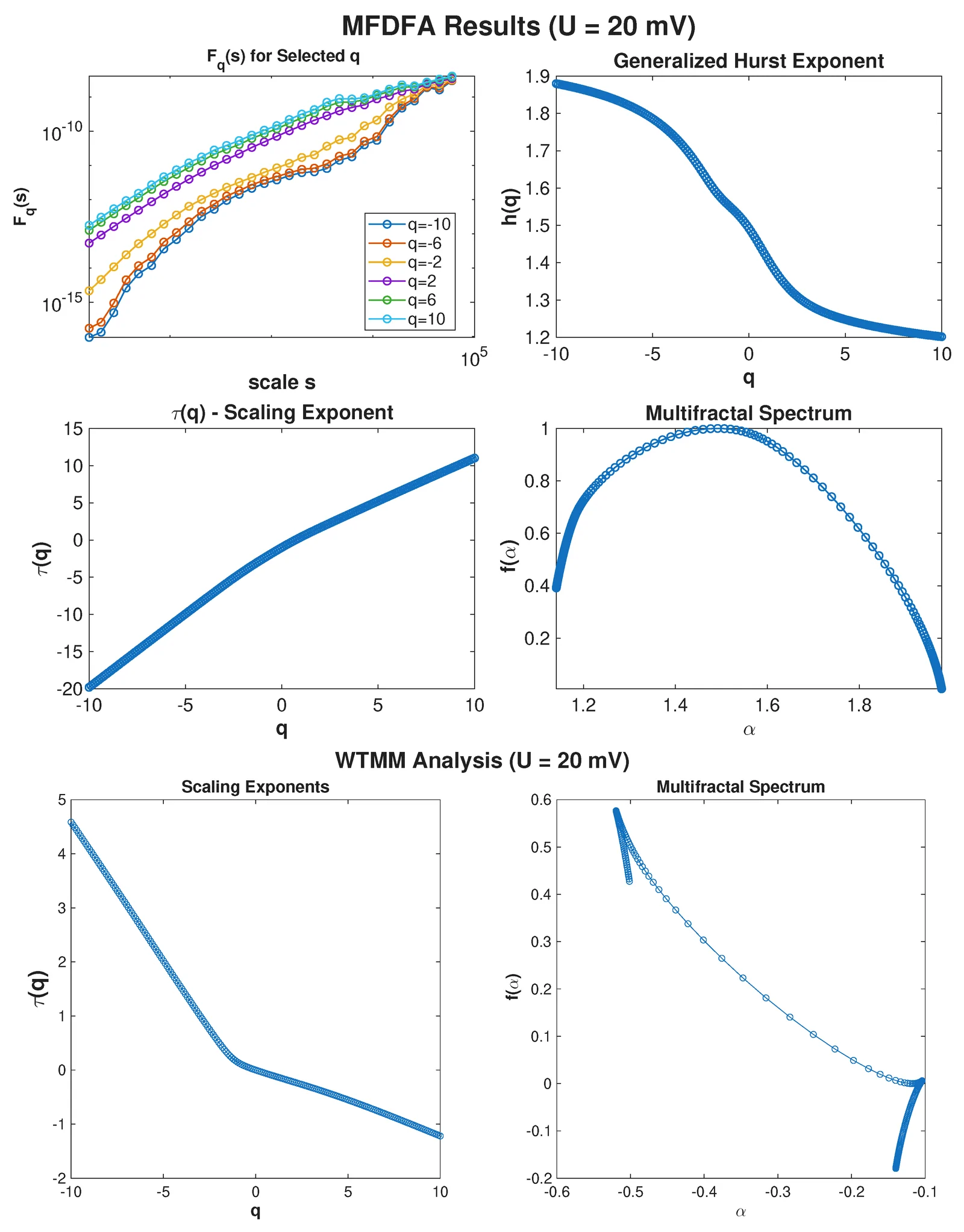
Control environment, 20 mV external potential. MFDFA vs. WTMM.

**Figure 4:**
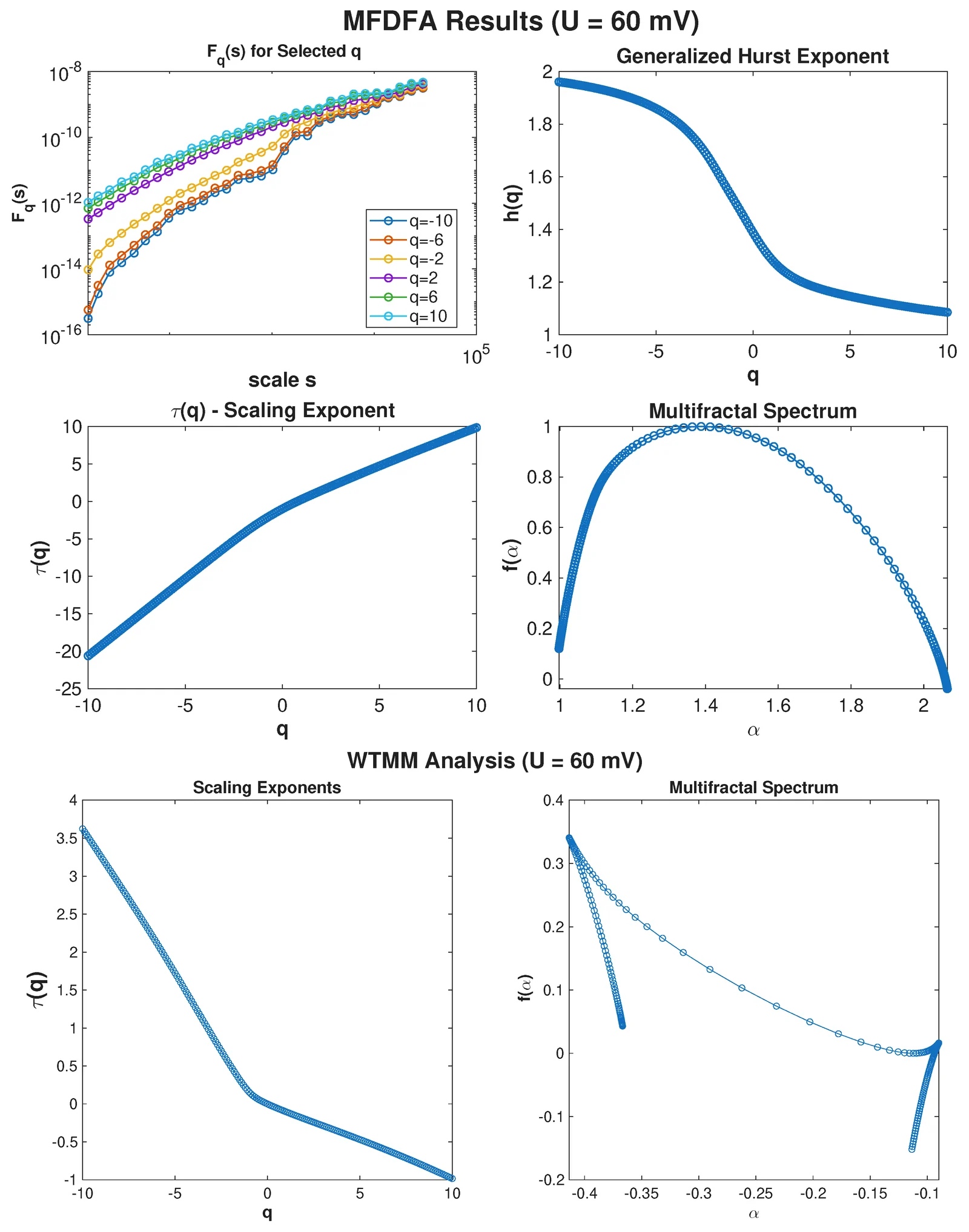
Control environment, 60 mV external potential. MFDFA vs. WTMM.

**Figure 5:**
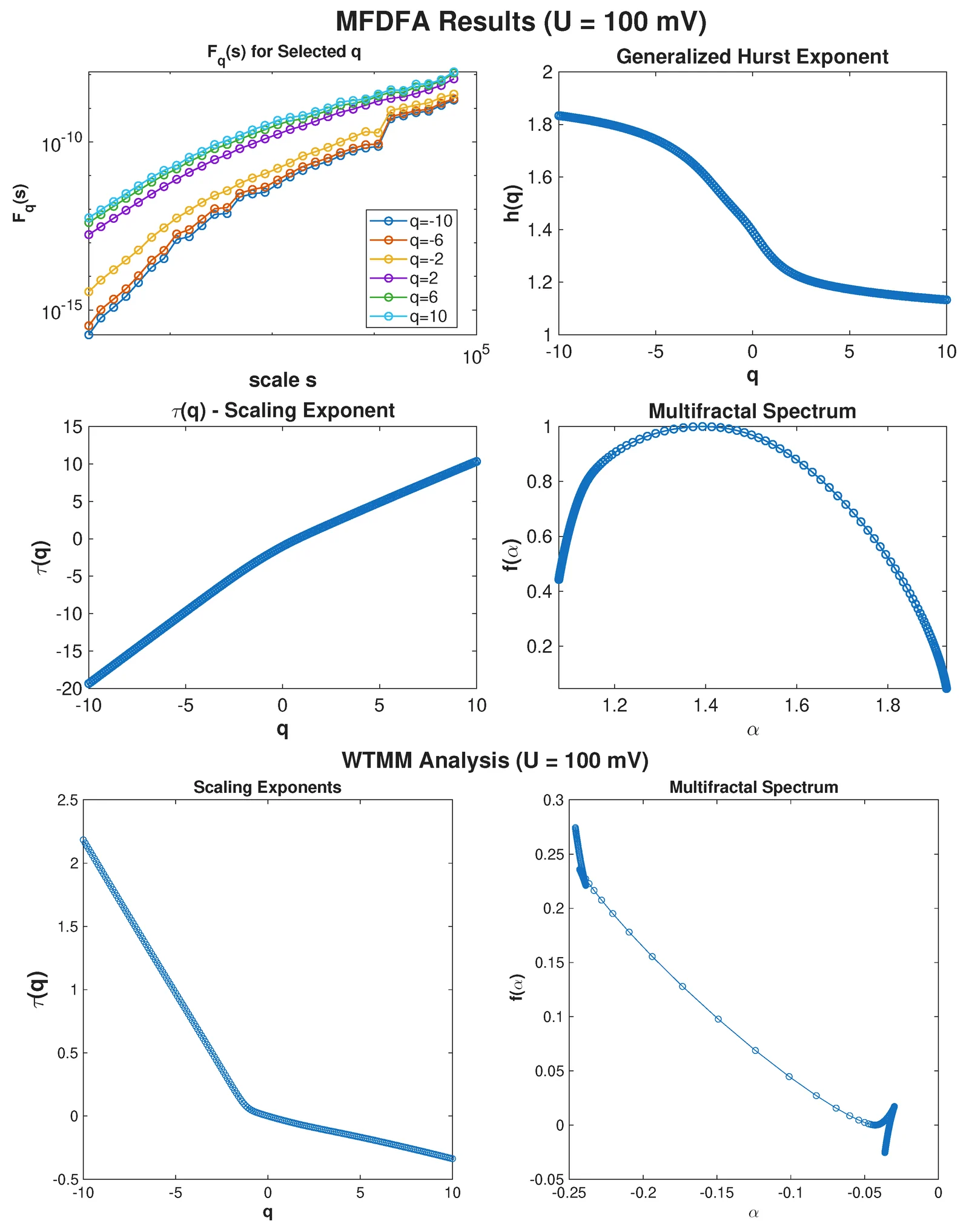
Control environment, 100 mV external potential. MFDFA vs. WTMM.

Naturally, the results presented in Figure 3, Figure 4 and Figure 5 show analysis of ion current recordings in different conditions, so it is obvious that there are many details to be analyzed, but firstly, fundamental difference characteristics for all the analyzed cases will be discussed.

Figure 3, Figure 4 and Figure 5 present the results of the multifractal analysis of ion-channel recordings by MFDFA (the four top figures are entitled: $F_{q}\left(s\right)$ for selected *q*, generalized Hurst exponent, scaling exponent (*q*), multifractal spectrum) and WTMM analysis (scaling exponents, multifractal spectrum). The key graphs showing multifractal features are the scaling exponents and multifractal spectrum. However, we would like to begin the analysis with the first graph of the MFDFA procedure, i.e., $F_{q}\left(s\right)$ for selected *q*. This graph presents examples of the fluctuations observed at different scales for a chosen power value “*q*”. As presented in Section 3.1, these graphs are fundamental for the MFDFA algorithm because the Hurst exponent is obtained as the slope of the linear function fitted to this data. The same pattern is observed at all presented examples (Figure 3, Figure 4 and Figure 5)—the relationship is not strictly linear. Although the quality of fit can be considered satisfactory from a statistical point of view, one can notice a systematic deviation from linearity. This picture is typical of all analyzed examples, not only those presented explicitly. The generalized Hurst exponents obtained on the basis of fitted linear functions are converted to the scaling exponent plot by Equation (5). The scaling exponent graphs are presented for both methods: MFDFA and WTMM. Besides clear differences in magnitude and convexity, they also show the existence of two correlation regimes: as q≈0, the slope of τ(q) changes. However, at WTMM methods, the change is more distinct as there are two clearly linear regimes with different slope coefficients. Analogously, the multifractal spectrum plots from both methods differ from a monofractal graph; the WTMM methods yield a more complex picture, and this finding is characteristic of all analyzed ion-channel time series. Therefore, it can be concluded that WTMM analysis provides more detailed information on the multifractal properties of ion-channel current time series.

Summarizing the key features of the WTMM results presented in Figure 3, Figure 4 and Figure 5, it can be noticed that the scaling exponents τ(q) and multifractality spectrum f(α) exhibit a clear nonlinear dependence on the moment order *q*, providing unambiguous evidence of multifractal behavior. However, both the curvature and the range of τ(q) show a pronounced voltage dependence. The analysis of the WTMM outcome is presented in the next section.

### 4.3. Statistical Comparison of Multifractal Spectrum Width Obtained Using MFDFA and WTMM

#### 4.3.1. Comparative Statistical Framework

The width of the multifractal spectrum (Δα) obtained using multifractal detrended fluctuation analysis (MFDFA) and Wavelet Transform Modulus Maxima (WTMM) was compared for each analyzed voltage (U=20,40,60,80,100 mV). Since both methods were applied to the same recordings, a paired statistical approach was used.

Differences between methods were assessed using the Wilcoxon signed-rank test [32], a non-parametric test suitable for paired observations and small sample sizes. The magnitude of the observed differences was quantified using the rank-biserial correlation (*RBC*) [33], which is a recommended effect-size measure for the Wilcoxon signed-rank test.

The rank-biserial correlation ranges from −1 to 1:

- |RBC|≈0.10—small effect;
- |RBC|≈0.30—moderate effect;
- |RBC|≈0.50—large effect;
- |RBC|>0.70—very large effect.

An RBC value of 1 indicates perfect separation between the compared conditions, meaning that all paired observations differ in the same direction.

#### 4.3.2. Results of Comparative Statistical Analysis

The Wilcoxon signed-rank test of multifractal spectrum widths (Δα) obtained using MFDFA and WTMM presented in Table 1 shows statistically significant differences for all investigated voltages (p<0.01). The average difference ranged from approximately 0.40 to 0.51 units of spectrum width.

The results of comparison between MFDFA and WTMM obtained from membranes which were incubated with IAA for 1 min are presented in Table 2. For MFDFA, the multifractal spectrum width increased from 0.764 at 20 mV to approximately 0.93 at 60–80 mV, corresponding to an increase of nearly 21%. This trend indicates that increasing membrane voltage enhances the degree of multifractality and complexity of the ion-channel current fluctuations. The largest spectrum widths (Δα) were observed at 60 and 80 mV, suggesting that these voltages correspond to the most complex dynamical regime of channel activity. In contrast, WTMM produced substantially smaller values and showed only weak voltage dependence. The spectrum width remained approximately constant over the investigated voltage range, varying between 0.329 and 0.382.

Analogously to already discussed cases, control environments and 1 min incubation with IAA, the Wilcoxon signed-rank test (Table 3) demonstrated highly significant differences between MFDFA and WTMM estimates for all investigated voltages (p<0.001). The average difference between methods ranged from 0.367 to 0.464 units of multifractal spectrum width.

The Wilcoxon signed-rank test in Table 4 reveals statistically significant differences between MFDFA and WTMM estimates of multifractal spectrum width at every investigated voltage (p<0.005). The average difference between methods ranged from 0.303 to 0.529. Compared with the 1 min and 5 min datasets, the 15 min incubation period produced a more pronounced voltage-dependent maximum at 80 mV, indicating that prolonged IAA exposure may enhance the voltage sensitivity of multifractal channel dynamics.

#### 4.3.3. Statistical Implications of the Results

The statistical analyses demonstrated that the differences between MFDFA and WTMM were highly significant for all investigated voltages and incubation times. For control recordings as well as for 1, 5, and 15 min IAA treatments, the paired Wilcoxon signed-rank test consistently yielded significant results (p<0.01), indicating that the observed discrepancies between the two methods cannot be explained by random variability alone. Furthermore, the rank-biserial correlation reached its maximum theoretical value (RBC=1.0) in all comparisons. This result indicates complete directional consistency among paired observations, meaning that every individual recording produced a larger multifractal spectrum width when analyzed using MFDFA than when analyzed using WTMM. The combination of highly significant *p*-values and maximal effect sizes demonstrates that the differences between the two multifractal approaches are not only statistically significant but also practically important. Consequently, the selection of the multifractal analysis method has a substantial impact on the quantitative estimation of multifractality in ion-channel current recordings. Importantly, the persistence of maximal effect sizes across all incubation times suggests that the systematic discrepancy between MFDFA and WTMM originates from the mathematical properties of the estimators rather than from biological variability induced by auxin treatment. In this point it should be recalled that the WTMM methods with the Haar wavelet, in principle, is better adjusted to the internal features of ion channels. However, the presented results should be considered as an indicator for further analysis.

## 5. Surrogate Analysis

To investigate the origin of multifractality, surrogate testing based on random shuffling of the time series was performed. Shuffling preserves the amplitude distribution while removing temporal correlations. Therefore, a significant reduction in the multifractal spectrum width after shuffling indicates that multifractality is primarily associated with long-range temporal organization. The results were obtained for 20 permutations of the measured ion-channel series.

The MFDFA results (presented in Table 5) reveal a strong reduction in the multifractal spectrum width after shuffling for every investigated membrane voltage. The reduction ranges from approximately 60% to 86%, demonstrating that the multifractality detected by MFDFA is strongly associated with temporal correlations. The largest reductions are generally observed at intermediate and high voltages (60–80 mV), suggesting that voltage-dependent channel dynamics contribute significantly to the long-range temporal organization of the current fluctuations.

Unlike MFDFA, WTMM (Table 6) exhibits only minor changes after shuffling. The average effect remains close to zero for all voltages and incubation times. In several cases, the shuffled signals even produce slightly larger spectrum widths than the original recordings.

The results demonstrate that membrane voltage plays an important role in shaping the multifractal structure of ion-channel currents. For MFDFA, the strongest multifractal behavior is typically observed at moderate and high voltages, where channel activity is expected to be most pronounced. Furthermore, the surrogate analysis shows that the voltage dependence detected by MFDFA largely disappears after destroying temporal correlations. This finding indicates that membrane voltage affects not only the amplitude of current fluctuations but also their temporal organization. In contrast, WTMM remains largely insensitive to shuffling throughout the entire voltage range. This observation suggests that WTMM primarily captures local singular structures that remain preserved after randomization of the temporal sequence. The consistent differences between the two methods indicate that MFDFA and WTMM probe different aspects of signal complexity. MFDFA appears to be mainly sensitive to voltage-dependent long-range temporal correlations, whereas WTMM predominantly reflects local irregularities of the signal.

## 6. WTMM Analysis of the Signal Voltage and IAA Incubation Influence

**Figure 6:**
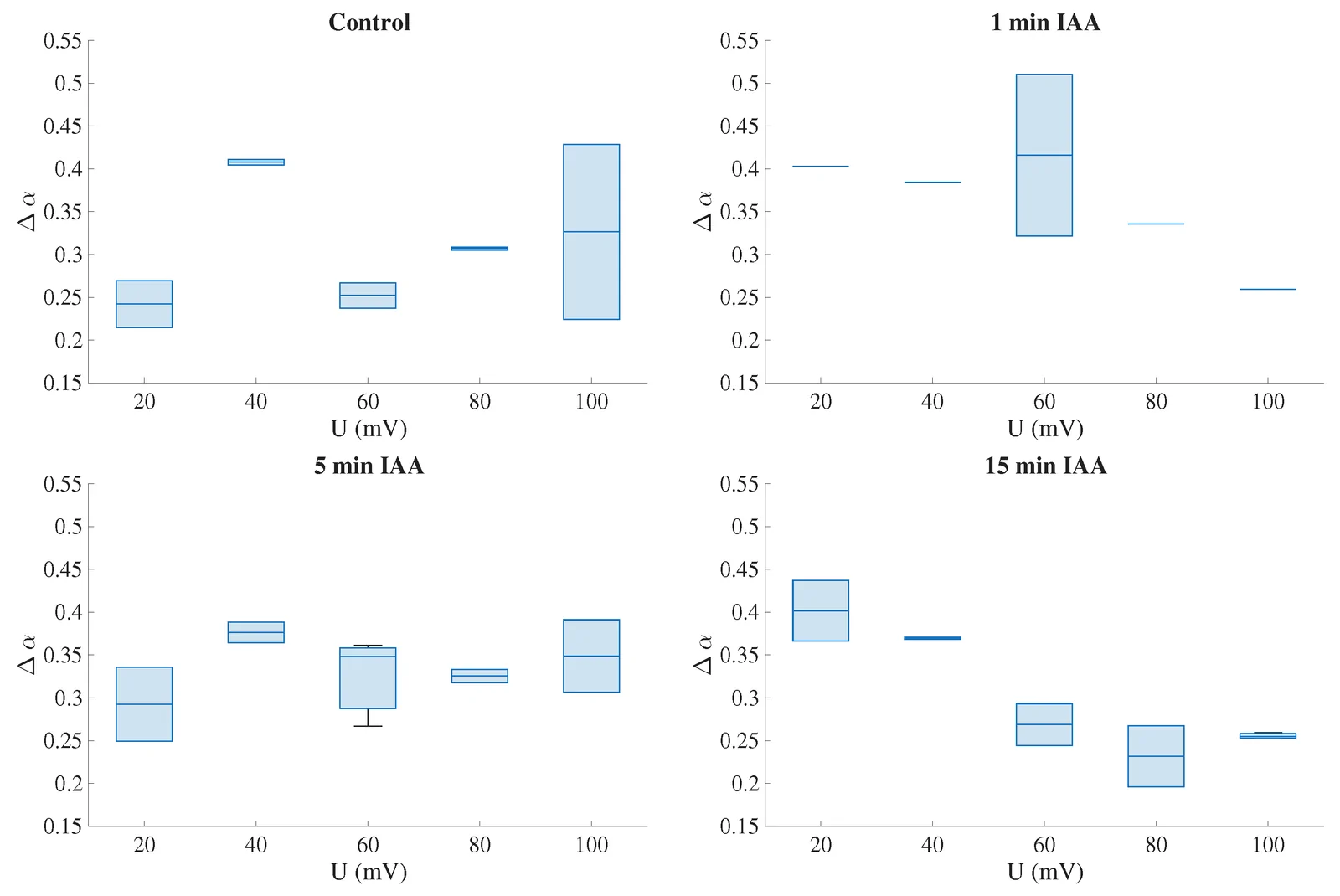
Boxplots of the observed multifractal spectrum widths versus membrane voltage for different incubation times. One active channel.

**Figure 7:**
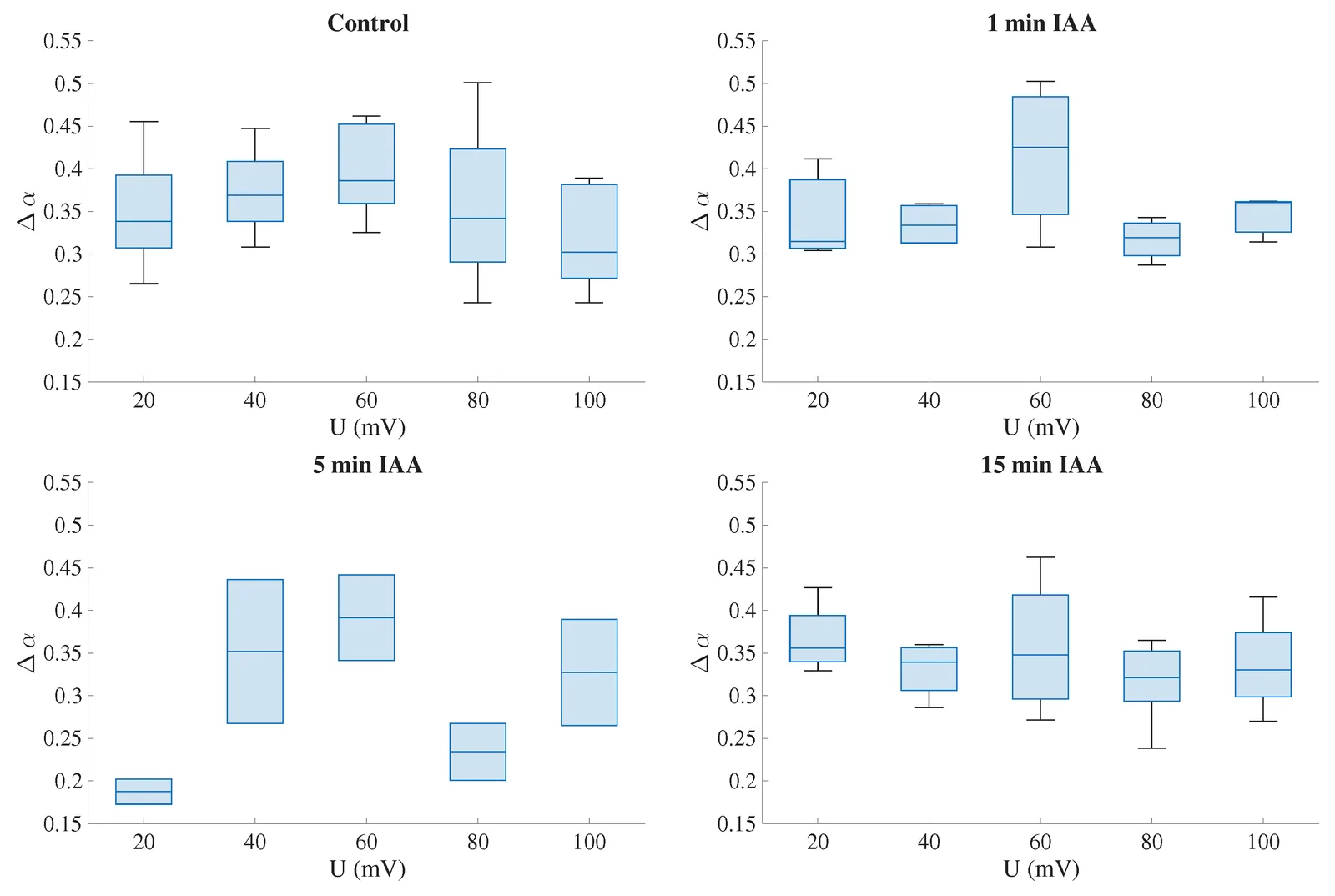
Boxplots of the observed multifractal spectrum widths versus membrane voltage for different incubation times. More than one active channel.

The multifractal spectrum provides detailed information on the fractal properties. The dynamical heterogeneity of the recorded signal is described by the width of the multifractal singularity spectrum; see Equation (11). The distribution of Δα was visualized for discrete membrane potentials (*U* = 20, 40, 60, 80, 100 mV) using MATLAB boxcharts for four experimental conditions: control (baseline membrane environment) and incubation in IAA for 1 min, 5 min, and 15 min for a single active channel (Figure 6) and two to three active channels (Figure 7).

(11) $$\Delta \alpha =\alpha_{max}-\alpha_{min}$$

The size of the spectrum range is considered a quantitative measure of heterogeneity in ion-channel gating dynamics. The experimental data have been grouped according to the number of active channels observed. The descriptive statistics plot of the spectrum range for the single active channels is presented in Figure 6, while the spectrum range boxplot for two or three active channels is presented in Figure 7.

### 6.1. Single-Channel Time Series

The main difficulty with one-channel recordings is the limited number of appropriate experimental data. The results presented in Figure 6 are based on one to three observations (Table 7). Therefore, it was not possible to apply any hypothesis testing methods. The presented analysis below is a qualitative observation only.

Across voltages, the boxchart panels (Figure 6) showed condition-dependent shifts in the distribution of Δα. The IAA 1 min panel spanned higher values (approximately 0.25–0.55) than the control panel (approximately 0.20–0.45), suggesting an early enhancement of multifractal width relative to baseline. In contrast, the IAA 5 min panel covered a narrower, lower range (approximately 0.24–0.40), consistent with a partial compression of Δα relative to 1 min. The IAA 15 min panel extended further downward (approximately 0.15–0.45), indicating that prolonged incubation can yield smaller Δα values than typically observed in the control condition.

However, it can be noticed that the presence of IAA may suppress the range of the multifractal spectrum. The 15 min incubation of the membrane in IAA results in a highly monotonic relationship between the spectrum range Δα and the membrane voltage. The membrane voltage is closely related to channel activity, so the presence of IAA narrows the range of the multifractal spectrum. A similar observation would be possible for 1 min incubation, but in this case, only one recording for the potential *U* = 20 mV, 40 mV, 80 mV, 100 mV, which makes this observation less valuable.

The visual trends were supported by the median and interquartile range, IQR of Δα values (Table 7). At 20 mV, the median Δα was high for IAA 1 min and IAA 15 min (∼0.40), exceeding IAA 5 min and control. At 60 mV, IAA 1 min showed the largest median and IQR, while control showed the smallest median and a tight IQR, consistent with increased heterogeneity at early incubation. At higher voltages (80–100 mV), the IAA 15 min medians were among the lowest, indicating a late-stage tendency toward reduced multifractal width. Importantly, the ranking of conditions varied with voltage (e.g., at 40 mV the control median exceeded IAA 5 min and IAA 15 min), highlighting voltage dependence of the incubation effect.

### 6.2. Two or Three Active Channels

The recordings in which two to three channels were simultaneously open were analyzed next. The presence of several ion channels in a limited space allows us to pose the question of the mutual interaction of the channels. Distributions of Δα were summarized at *U* = 20, 40, 60, 80, 100 mV for control and for IAA incubation times of 1 min, 5 min, and 15 min (Figure 7).

The distributions (Figure 7) suggest voltage-dependent modulation of Δα by IAA. The median of Δα for the control environment is increasing up to *V* = 60 mV, then decreasing. This is correlated with the typical activity of an ion channel, which is induced by the external membrane potential. Increasing the voltage increases channel activity. This is correlated with the increase in the width of the multifractal spectrum. At *U* = 60 mV it achieves maximum. An increase in external potential increases the channel’s activity, i.e., the time it spends in the open state, thereby suppressing the role of background noise and decreasing the width of the spectrum. This effect is suppressed in the presence of IAA, particularly in 1 min and 15 min of incubation. The 5 min condition tends to shift Δα toward smaller values at low and intermediate voltages, whereas 1 min and 15 min often remain closer to the control range.

The descriptive statistics (Table 8) confirm that the effect of incubation depends on voltage. At 20 mV, the median Δα decreases markedly in IAA 5 min compared with the other conditions, while IAA 15 min shows a median comparable to or slightly above control. At 60 mV, the IAA 1 min condition exhibits the largest median and a relatively broad IQR, whereas IAA 15 min returns toward control-like values. At 100 mV, medians in IAA 1 min and IAA 15 min exceed the control median, suggesting that early and late incubation can increase multifractal width at high voltage.

A comparison of the multifractal spectrum width Δα between single-channel (Figure 6, Table 7) and multi-channel (two or three simultaneously active channels; Figure 7, Table 8) recordings reveals systematic differences that indicate the presence of collective effects beyond single-channel stochastic dynamics.

The influence of IAA differs between the two regimes. In the single-channel case, IAA tends to reduce Δα, particularly at longer incubation times, indicating a suppression of dynamical heterogeneity. For multiple channels, however, the effect is more complex and strongly voltage-dependent. The 5 min incubation typically shifts Δα toward lower values at low and intermediate voltages, whereas the 1 min and 15 min conditions often remain comparable to or exceed the control values. This behavior indicates that IAA affects not only single-channel kinetics but also the collective dynamics of multiple channels.

The persistence of a broad multifractal spectrum after channel aggregation suggests that the observed scaling properties cannot be interpreted as a simple consequence of summing statistically independent channel currents (Equation (12)).

(12) $$I\left(t\right)=\sum_{k=1}^{N}i_{k}\left(t\right)$$

$i_{k}\left(t\right)$ represents independent channel currents.

If the total current were merely the superposition of independent stochastic processes, averaging effects associated with increasing channel number would be expected to reduce fluctuation heterogeneity and progressively narrow the multifractal spectrum. Such behavior is consistent with general scaling arguments and with previous studies showing that random mixing and averaging tend to suppress multifractal signatures and drive the signal toward more homogeneous statistical behavior [20]. The fact that pronounced multifractality is preserved, together with the emergence of a systematic voltage dependence of the multifractal parameters, suggests the presence of additional mechanisms beyond independent channel activity. These mechanisms may involve long-range temporal correlations, memory effects associated with conformational dynamics, or collective interactions occurring across multiple temporal scales. From this perspective, multifractality appears to reflect intrinsic dynamical properties of ion-channel gating rather than being solely a statistical consequence of channel superposition [13,16].

## 7. Conclusions

The results obtained using both the multifractal detrended fluctuation analysis (MFDFA) and the Wavelet Transform Modulus Maxima (WTMM) methods consistently demonstrate that ion-channel current recordings exhibit clear multifractal properties. However, contrary to the common assumption that both approaches provide interchangeable estimates of multifractal characteristics, our results reveal statistically significant differences between the multifractal parameters obtained with the two methods, as demonstrated in Section 4.3. These findings indicate that the estimated multifractal properties are influenced not only by the intrinsic structure of the analyzed signal but also by the methodological framework used for their characterization. Moreover, the surrogate analysis in Section 5 demonstrates that the multifractality detected by MFDFA is strongly voltage-dependent and largely originates from long-range temporal correlations. Across all voltages and incubation times, shuffling reduces the multifractal spectrum width by approximately 60–86%. Conversely, WTMM exhibits almost no systematic response to signal randomization, suggesting that it captures multifractal properties that are largely independent of temporal ordering. These findings indicate that the multifractal organization of ion-channel currents cannot be explained solely by broad amplitude distributions. Instead, it arises from a complex interplay between membrane voltage, long-range temporal correlations, and local signal singularities. From a broader perspective, the most important conclusion of this study is that future efforts should focus primarily on understanding the physical and biological origins of multifractality in ion-channel dynamics, as well as the functional significance of this property. The identification of multifractal scaling behavior alone does not explain the mechanisms responsible for its emergence. A deeper understanding of the stochastic gating processes, channel conformational dynamics, and long-range temporal correlations underlying ion transport is necessary before definitive conclusions regarding the biological role of multifractality can be drawn.

The present results demonstrate that MFDFA and WTMM cannot be considered interchangeable techniques for quantitative multifractal characterization of ion-channel current fluctuations. The WTMM method is particularly well suited for analyzing ion-channel currents, outperforming the MFDFA approach across several key quantitative metrics. The key result is that WTMM provides a more faithful representation of the signal’s intrinsic structure by directly probing local singularities, which is essential for ion-channel recordings characterized by abrupt transitions between open and closed states. In contrast, the MFDFA results exhibit systematic deviations from linear scaling in the $F_{q}\left(s\right)$ plots (Figure 3, Figure 4 and Figure 5), indicating imperfect power-law behavior and introducing uncertainty in the estimation of the generalized Hurst exponent h(q).

The superiority of the WTMM is also manifested through a clear quantitative difference in the curvature of the scaling exponent function τ(q). The WTMM method yields a strongly nonlinear τ(q) with well-defined slope changes, revealing distinct scaling regimes, particularly near q≈0. In comparison, the MFDFA-derived τ(q) is smoother and less structured, indicating reduced sensitivity to changes in correlation properties across fluctuations of different magnitudes. This difference translates directly into the multifractal spectrum f(α), where WTMM produces broader and more asymmetric spectra, reflecting a richer hierarchy of singularities. Moreover, the scaling ranges are more clearly identifiable within the WTMM framework. The wavelet-based approach allows for a more robust separation of scaling regimes across scales, whereas MFDFA relies on fitting procedures over ranges where residual trends and finite-size effects may bias the estimates. As a consequence, the WTMM-derived exponents exhibit greater stability and interpretability, particularly for nonstationary signals. WTMM also shows greater sensitivity to dynamical changes, as quantified by the width of the multifractal spectrum, Δα. Variations in Δα with membrane voltage and IAA incubation are more pronounced and systematically structured in the WTMM results, enabling the detection of subtle transitions such as the emergence of a maximum at intermediate voltages or the suppression of multifractality under biochemical modulation. In contrast, the corresponding MFDFA estimates are less responsive and tend to underestimate the degree of multifractal variability.

The WTMM analysis was based on the Haar wavelet. This choice was motivated by the dichotomous nature of ion-channel recordings, which are characterized by abrupt transitions between discrete conductance states. The Haar wavelet provides excellent localization of discontinuities and singular events and is therefore particularly well suited for detecting the abrupt opening and closing transitions that dominate patch-clamp recordings. Moreover, Haar wavelets have been widely used in the analysis of signals with piecewise-constant or step-like behavior due to their capability to preserve local discontinuities and sharp temporal changes [34]. Nevertheless, the question of the optimal analyzing wavelet remains open. Although the Haar wavelet appears to be a physically justified choice for ion-channel signals, a rigorous demonstration of its optimality relative to other wavelet families has not yet been established. In particular, wavelets possessing higher numbers of vanishing moments, such as derivatives of Gaussian wavelets commonly employed in continuous WTMM analysis, may emphasize different aspects of signal regularity and could potentially yield alternative estimates of multifractal spectra [13,16]. The observed discrepancies between MFDFA and WTMM further suggest that methodological factors may substantially influence the quantitative description of multifractality. Consequently, future studies should first aim to elucidate the biological mechanisms responsible for the multifractal organization of ion-channel fluctuations and determine whether multifractal behavior reflects specific physiological processes involved in channel regulation and transport. Once these mechanisms are better understood, methodological choices—including the selection of the mother wavelet and multifractal analysis framework—can be optimized in a more informed and physically meaningful manner. This issue constitutes an important direction for future research.

The observed discrepancies between MFDFA and WTMM highlight the need for further investigations aimed at identifying the physical origin of multifractality and determining how voltage-dependent channel dynamics contribute to the emergence of multiscale behavior in ion-channel currents.

## Figures and Tables

**Table 1:** Comparison of multifractal spectrum widths (Δα) obtained using MFDFA and WTMM. The mean values are presented for both methods. The control environment.

*U* (mV)	*N*	MFDFA	WTMM	Difference	*p*-Value	RBC
20	12	0.726	0.325	0.402	0.00049	1.000
40	12	0.804	0.332	0.471	0.00049	1.000
60	13	0.783	0.372	0.411	0.00024	1.000
80	12	0.835	0.325	0.510	0.00049	1.000
100	10	0.825	0.326	0.499	0.00195	1.000

**Table 2:** Comparison of multifractal spectrum widths (Δα) obtained using MFDFA and WTMM. The mean values are presented for both methods. Incubation time with IAA: 1 min.

*U* (mV)	*N*	MFDFA	WTMM	Difference	*p*-Value	RBC
20	16	0.764	0.329	0.435	$3.05\times 10^{-5}$	1.000
40	13	0.832	0.382	0.450	$2.44\times 10^{-4}$	1.000
60	13	0.924	0.350	0.574	$2.44\times 10^{-4}$	1.000
80	12	0.928	0.346	0.581	$4.88\times 10^{-4}$	1.000
100	13	0.856	0.332	0.524	$2.44\times 10^{-4}$	1.000

**Table 3:** Comparison of multifractal spectrum widths (Δα) obtained using MFDFA and WTMM. The mean values are presented for both methods. Incubation time with IAA: 5 min.

*U* (mV)	*N*	MFDFA	WTMM	Difference	*p*-Value	RBC
20	12	0.698	0.331	0.367	$4.88\times 10^{-4}$	1.000
40	12	0.716	0.345	0.371	$4.88\times 10^{-4}$	1.000
60	13	0.739	0.357	0.383	$2.44\times 10^{-4}$	1.000
80	12	0.810	0.346	0.464	$4.88\times 10^{-4}$	1.000
100	14	0.736	0.328	0.408	$1.22\times 10^{-4}$	1.000

**Table 4:** Comparison of multifractal spectrum widths (Δα) obtained using MFDFA and WTMM. The mean values are presented for both methods. Incubation time with IAA: 15 min.

*U* (mV)	*N*	MFDFA	WTMM	Difference	*p*-Value	RBC
20	9	0.685	0.382	0.303	0.00391	1.000
40	10	0.731	0.340	0.390	0.00195	1.000
60	12	0.651	0.327	0.325	0.00049	1.000
80	11	0.841	0.312	0.529	0.00098	1.000
100	15	0.729	0.317	0.412	0.00006	1.000

**Table 5:** Average multifractal spectrum width obtained using MFDFA for original and shuffled signals.

Voltage (mV)	Control (%)	IAA 1 min (%)	IAA 5 min (%)	IAA 15 min (%)
20	71.1	73.0	86.3	69.0
40	76.2	77.9	74.8	60.5
60	84.1	81.8	75.0	65.2
80	80.1	78.9	84.8	72.2
100	71.4	74.4	72.7	68.3

**Table 6:** Average effect of shuffling on WTMM multifractal spectrum width.

Voltage (mV)	Control (%)	IAA 1 min (%)	IAA 5 min (%)	IAA 15 min (%)
20	−7.2	−7.2	5.5	23.2
40	13.3	2.3	10.9	10.0
60	−0.7	17.1	2.4	−15.5
80	−11.3	0.0	8.5	−15.0
100	−3.1	6.3	−1.0	−1.4

**Table 7:** Width of the multifractal singularity spectrum, Δα, summarized as median [Q1–Q3] (min–max) with sample size *N* for each membrane potential *U* and incubation time.

*U* (mV)	Control	IAA 1 min	IAA 5 min	IAA 15 min
20	0.242, *N* = 2	0.403, *N* = 1	0.292, *N* = 2	0.402, *N* = 2
	[0.228–0.256]	[0.403–0.403]	[0.271–0.314]	[0.384–0.419]
	(0.215–0.270)	(0.403–0.403)	(0.249–0.335)	(0.366–0.437)
40	0.408, *N* = 2	0.384, *N* = 1	0.376, *N* = 2	0.369, *N* = 2
	[0.406–0.409]	[0.384–0.384]	[0.370–0.382]	[0.369–0.370]
	(0.405–0.411)	(0.384–0.384)	(0.364–0.388)	(0.368–0.371)
60	0.252, *N* = 2	0.416, *N* = 2	0.348, *N* = 3	0.269, *N* = 2
	[0.245–0.260]	[0.369–0.463]	[0.308–0.355]	[0.257–0.281]
	(0.237–0.267)	(0.322–0.511)	(0.267–0.361)	(0.244–0.293)
80	0.307, *N* = 2	0.336, *N* = 1	0.326, *N* = 2	0.232, *N* = 2
	[0.306–0.308]	[0.336–0.336]	[0.322–0.329]	[0.214–0.249]
	(0.305–0.309)	(0.336–0.336)	(0.318–0.333)	(0.196–0.267)
100	0.326, *N* = 2	0.259, *N* = 1	0.349, *N* = 2	0.255, *N* = 3
	[0.275–0.378]	[0.259–0.259]	[0.328–0.370]	[0.254–0.257]
	(0.224–0.429)	(0.259–0.259)	(0.307–0.391)	(0.252–0.259)

**Table 8:** Width of the multifractal singularity spectrum, Δα, for the 2–3 open-channel regime, summarized as median [Q1–Q3] (min–max) with sample size *N* for each membrane potential *U* and incubation time.

*U* (mV)	Control	IAA 1 min	IAA 5 min	IAA 15 min
20	0.338, *N* = 5	0.315, *N* = 3	0.188, *N* = 2	0.356, *N* = 4
	[0.321–0.371]	[0.309–0.363]	[0.180–0.195]	[0.345–0.377]
	(0.265–0.455)	(0.304–0.412)	(0.173–0.202)	(0.329–0.427)
40	0.369, *N* = 4	0.334, *N* = 4	0.352, *N* = 2	0.339, *N* = 4
	[0.353–0.389]	[0.313–0.356]	[0.309–0.394]	[0.316–0.354]
	(0.308–0.447)	(0.313–0.359)	(0.267–0.436)	(0.286–0.360)
60	0.386, *N* = 5	0.425, *N* = 4	0.392, *N* = 2	0.348, *N* = 4
	[0.371–0.449]	[0.365–0.475]	[0.366–0.417]	[0.309–0.396]
	(0.325–0.462)	(0.308–0.502)	[0.366–0.417]	[0.309–0.396]
80	0.342, *N* = 4	0.319, *N* = 4	0.234, *N* = 2	0.321, *N* = 5
	[0.315–0.384]	[0.304–0.333]	[0.217–0.251]	[0.312–0.348]
	(0.243–0.501)	(0.287–0.343)	(0.201–0.268)	(0.238–0.365)
100	0.302, *N* = 5	0.361, *N* = 3	0.327, *N* = 2	0.330, *N* = 4
	[0.281–0.379]	[0.338–0.361]	[0.296–0.358]	[0.313–0.353]
	(0.243–0.389)	(0.314–0.362)	(0.265–0.390)	(0.270–0.416)

## Data Availability

Data available on request.

## Abbreviations

The following abbreviations are used in this manuscript:

IAA	auxin
SV	slowly activated channel
DFA	detrended fluctuation analysis
MFDFA	multifractal detrended fluctuation analysis
WTMM	wavelet transform modulus maxima
